# Prediction of Adverse Maternal Outcomes in Preeclampsia Using the FullPIERS (Preeclampsia Integrated Estimate of Risk) Model in a Tertiary Care Hospital of Eastern India

**DOI:** 10.7759/cureus.66664

**Published:** 2024-08-12

**Authors:** Dipon Burman, Sanjukta Das, Jayeeta Burman, Sembagamuthu Sembiah

**Affiliations:** 1 Obstetrics and Gynaecology, Balurghat Super Specialty Hospital, Balurghat, IND; 2 Obstetrics and Gynaecology, Gangarampur Super Specialty Hospital, Gangarampur, IND; 3 Community Medicine, Sarat Chandra Chattopadhyay Government Medical College & Hospital, Uluberia, IND; 4 Community Medicine and Family Medicine, All India Institute of Medical Sciences, Kalyani, Kalyani, IND

**Keywords:** preeclampsia, fullpiers model, predictive tools, risk assessment, adverse maternal outcomes, pre-eclampsia

## Abstract

Introduction: Preeclampsia, characterized by hypertensive disorders and systemic inflammatory response, remains a leading cause of maternal morbidity and mortality globally. Effective risk assessment tools are crucial for predicting adverse maternal outcomes.

Objective: This study evaluates the performance of the fullPIERS (Preeclampsia Integrated Estimate of Risk) model in predicting adverse maternal outcomes within 24 hours of admission for preeclampsia.

Methods: A cross-sectional study was conducted over one year, involving 100 preeclamptic patients admitted to Nil Ratan Sircar Medical College & Hospital (NRSMCH). Predictor variables were collected within 24 hours of admission and analyzed using the fullPIERS model.

Results: The fullPIERS model effectively stratified maternal risk. Adverse outcomes were significantly associated with systolic blood pressure (BP) ≥ 140 mmHg, diastolic BP ≥ 90 mmHg, oxygen saturation ≤ 95%, frontal headache, visual disturbances, chest pain/dyspnea, and abnormal random blood sugar, albumin, alanine aminotransferase, platelet count, and creatinine levels. A fullPIERS score ≥ 30 was strongly predictive of adverse maternal outcomes.

Conclusion: The fullPIERS model is a valuable tool for predicting adverse maternal outcomes in preeclampsia, aiding in timely and effective clinical decision-making.

## Introduction

Preeclampsia, characterized by hypertensive disorders and systemic inflammatory response, remains a leading cause of maternal morbidity and mortality globally [1]. The World Health Organization estimates that preeclampsia results in over 76,000 maternal deaths annually, translating to the loss of one woman every seven minutes globally [2]. Furthermore, beyond these recorded fatalities, many women experience near-miss maternal morbidity, ranging from mild asymptomatic hypertension to severe neurological, renal, and cardiopulmonary compromise [3]. Timely identification and treatment of preeclampsia are imperative for ensuring favorable maternal and perinatal outcomes. However, despite advances in obstetric care, outcomes remain less favorable in women residing in developing countries, underscoring disparities in healthcare access and quality [4]. The definitive treatment for preeclampsia lies in the termination of pregnancy, but this poses challenges, especially for preterm fetuses who face heightened risks of morbidity and mortality [5]. Consequently, clinicians often employ supportive and temporizing measures to improve perinatal outcomes, particularly when preeclampsia arises remotely from the term.

Yet, uncertainties persist regarding the balance between maternal risks associated with expectant management and its potential benefits, necessitating effective risk assessment tools. Existing risk assessment methods have demonstrated poor performance, highlighting the urgent need for validated tools capable of accurately stratifying maternal risk. Among these, the fullPIERS (Preeclampsia Integrated Estimate of Risk) model has emerged as a promising predictive tool, offering a systematic approach to risk assessment and management in preeclampsia. The fullPIERS model is a well-validated tool aimed at predicting adverse maternal outcomes in women with preeclampsia. This model uses a combination of clinical and laboratory parameters such as blood pressure (BP) readings, oxygen saturation (SPO2) levels, symptoms like headache and visual disturbances, and specific laboratory tests. By integrating these factors, the fullPIERS model generates a risk score that aids clinicians in identifying high-risk patients, thereby facilitating timely intervention and management [6].

This study aims to evaluate the performance of the fullPIERS model in predicting adverse maternal outcomes when predictor variables are obtained within 24 hours of admission for preeclampsia, thus bridging a crucial gap in risk assessment and management strategies for preeclamptic mothers remote from term or undergoing induction of labor. Through a comprehensive evaluation of predictive models and risk assessment tools, this research endeavors to enhance our understanding of preeclampsia and improve clinical decision-making, ultimately striving toward better outcomes for both mothers and babies.

## Materials and methods

This single-center, institution-based observational study employed a cross-sectional study design and was conducted over one year, spanning from April 1st, 2018, to March 31st, 2019.

Study participants

All participants were admitted as inpatients in the Department of Obstetrics and Gynecology at Nil Ratan Sircar Medical College & Hospital (NRSMCH). The study focused on the first 100 subjects admitted during the specified study period. Purposive sampling was used to select subjects who met the inclusion criteria for the study.

Participants were included if they were admitted with preeclampsia or had developed preeclampsia after admission. Preeclampsia was defined as (i) blood pressure ≥ 140/90 mmHg (at least one component, twice, ≥ four hours apart, after 20 weeks) and either proteinuria (of ≥2+ by dipstick, ≥0.3 g per day by 24-hour collection, or ≥30 mg/mmol by urinary protein-to-creatinine ratio) or hyperuricemia (greater than the local upper limit of local non-pregnancy normal range) [7]; (ii) HELLP (hemolysis, elevated liver enzymes, low platelets) syndrome, even in the absence of hypertension or proteinuria; or (iii) superimposed preeclampsia (rapidly increasing requirements for antihypertensive drugs, systolic blood pressure >170 mmHg or diastolic blood pressure >120 mmHg, new proteinuria, or new hyperuricemia) [8]. This definition, although differing from many international definitions, reflects both the variable and multisystem nature of preeclampsia at presentation and the range of women seen in clinical practice. Subjects were excluded if they were admitted in spontaneous labor or had experienced any component of adverse maternal outcomes before becoming eligible or before data collection began. This approach ensured that the study focused on a specific subset of preeclamptic patients, providing a clearer understanding of the predictive power of the fullPIERS model for adverse maternal outcomes. Data collection relied solely on parameters obtained within 24 hours of admission.

Study variables

The fullPIERS model is a predictive tool used to assess the risk of adverse maternal outcomes in women with preeclampsia. It combines several clinical, laboratory, and demographic variables to generate a risk score. The exact formula for calculating the fullPIERS score is based on a logistic regression model that has been validated in clinical studies [9]. The fullPIERS model equation typically takes the form of a logistic regression equation, which looks like this:

Logit (P) = β0 + β1 (gestational age) + β2 (systolic BP) + β3 (diastolic BP) + β4 (SPO2) + β5 (serum creatinine) + β6 (serum uric acid) + β7 (serum platelets) + β8 (serum aspartate aminotransferase) + β9 (age) + β10 (parity) + β11( headache) + β12 (visual disturbances) + β13 (chest pain/ dyspnea); where β0 is the intercept, and β1-β13 are the coefficients for each predictor variable [6].

Adverse maternal outcomes were either maternal mortality or one or more serious CNS, cardiorespiratory, hepatic, renal, or hematological morbidity.

Ethical approval

Ethical approval was obtained from the Institutional Ethics Committee of Nil Ratan Sircar Medical College & Hospital, vide no: NMC/450. Informed written consent was taken from all participants, ensuring compliance with ethical principles and guidelines for human subjects research.

Data analysis

Data were entered in Microsoft Excel 2007 (Microsoft Corporation, Redmond, WA) and analyzed using the SPSS software version 16.0 (SPSS Inc., Chicago, IL). The association between individual study variables, as well as the fullPIERS score, and adverse maternal outcomes, were analyzed using logistic regression analysis, with a significance level set at P < 0.05.

## Results

The study included 100 preeclamptic patients admitted to NRSMCH. Significant findings revealed that the majority of the subjects (37%) were aged between 21 and 25 years, most were primigravid (64%), and had zero parity (70%). Gestational age at admission varied, with 36% admitted between 29 and 34 weeks. Almost all participants (99%) had no history of smoking or gestational diabetes. Significant clinical signs at admission included elevated systolic BP (≥140 mmHg) in 82% and elevated diastolic BP (≥90 mmHg) in 57% of patients. Lower SPO2 levels (≤95%) were observed in 28% of patients, and 52% had an abdominal circumference below the 5th percentile. Biochemical analysis revealed that 67% had abnormal uric acid levels.

Among the participants, 34% had adverse maternal outcomes. The specific adverse outcomes observed included eclampsia in 18%; five cases of abruptio placentae and thrombocytopenia each; with two cases of acute renal failure, pulmonary edema, and postpartum hemorrhage each. Notably, there was no maternal mortality in our study cohort.

Significant predictors of adverse outcomes included abnormal random blood sugar levels (70.6% vs. 29.4%, p = 0.0001), abnormal albumin levels (54.5% vs. 45.5%, p = 0.021), and abnormal creatinine levels (93.3% vs. 6.7%, p = 0.0001). Elevated systolic BP (≥140 mmHg) and diastolic BP (≥90 mmHg), lower SPO2 levels (≤ 95%), and clinical symptoms such as frontal headache and visual disturbances were significantly associated with adverse outcomes (Table 1).

**Table 1:** Association of demographic, clinical, and laboratory parameters with adverse maternal outcomes (n = 100). BP: blood pressure; SPO2: oxygen saturation; ALT: alanine aminotransferase; AST: aspartate aminotransferase.

Predictors	Adverse maternal outcome (present) (n = 34)	Adverse maternal outcome (absent) (n = 66 )	Odds ratio (95% confidence interval); p-value
Age			
<30	11 (52.4)	10 (15.2)	1
≥30	23 (29.1)	56 (84.8)	2.6 (1.05-6.7); 0.04
Gestational age			
<34	12 (44.4)	15 (55.6)	1
≥34	22 (30.1)	51 (69.9)	1.8 (0.8-4); 0.135
Systolic BP			
≥140	32 (39)	50 (61)	5.1 (1.1-23.8); 0.02
<140	2 (11.1)	16 (88.9)	1
Diastolic BP			
≥90	24 (42.1)	33 (57.9)	2.4 (0.9-6.2); 0.3
<90	10 (23.3)	33 (76.7)	1
SPO2			
≤95	17 (60.7)	11 (39.3)	5 (1.7-14.1); 0.001
>95	17 (23.6)	55 (76.4)	1
Frontal headache			
Yes	14 (63.6)	8 (36.4)	5 (1.8-13.7); 0.001
No	20 (25.6)	58 (74.4)	1
Visual disturbance			
Yes	13 (68.4)	8 (36.4)	4.4 (1.6-12.2); 0.001
No	21 (25.9)	58 (74.4)	1
Chest pain/dyspnea			
Yes	9 (34.6)	1 (1.4)	38.6 (4.7-316);0.001
No	17 (65.4)	73 (98.6)	1
Random blood sugar			
Abnormal	12 (70.6)	5 (29.4)	6.6 (1.9-22.8); 0.001
Normal	22 (26.5)	61 (73.4)	1
Albumin			
Abnormal	12 (54.5)	10 (45.5)	2 (1.1-7.9); 0.0001
Normal	22 (28.2)	56 (71.8)	1
ALT			
Abnormal	20 (44.4)	25 (55.6)	2.3 (1-5.3); 0.05
Normal	14 (25.5)	41 (74.5)	1
AST			
Abnormal	10 (32.3)	21 (67.7)	0.8 (0.3-2.1); 0.4
Normal	24 (34.8)	45 (65.2)	1
Platelet			
Abnormal	25 (50)	25 (50)	4.5 (1.8-11.2); 0.0001
Normal	9 (18)	41 (82)	1
Creatinine			
Abnormal	14 (93.3)	1 (6.7)	7.4 (5-36.4); 0.0001
Normal	20 (23.5)	65 (76.5)	1

The relative risk (RR) of experiencing an adverse maternal outcome with a fullPIERS score of 30 or higher was found to be 5.5 (95% CI: 3.3-8.9), with a statistically significant p-value of 0.0001 (Table 2).

**Table 2:** Association between fullPIERS score and adverse maternal outcomes (n = 100). fullPIERS: Preeclampsia Integrated Estimate of Risk.

fullPIERS score	Adverse maternal outcome present, n (%)	Adverse maternal outcome absent, n (%)	Relative risk (95% confidence interval); p-value
≥30	21 (72.4)	8 (27.6)	5.5 (3.3-8.9); 0.001
<30	13 (18.3)	58 (81.7)	1

This suggests that a fullPIERS score of 30 or higher is strongly associated with an increased risk of adverse maternal outcomes, highlighting its potential utility in clinical practice for the early identification and management of high-risk preeclamptic patients.

## Discussion

The findings of this study shed light on the demographic, clinical, and biochemical characteristics of patients with preeclampsia, as well as their association with adverse maternal outcomes. By comparing these results with similar studies conducted in India and other countries, we can gain a deeper understanding of the factors influencing outcomes in this high-risk obstetric condition.

In comparing the demographic characteristics of our study population with those of similar studies conducted in India, we observed similarities in terms of maternal age distribution, gravidity, and parity [4,10]. However, variations may exist depending on the study population and setting. For instance, urban versus rural populations or tertiary care centers versus primary health facilities may yield different demographic profiles [10].

Regarding clinical symptoms and signs, our study revealed comparable findings to previous research conducted in India and other countries. The prevalence of symptoms such as headache, visual disturbances, and right upper quadrant pain aligns with existing literature on preeclampsia [11,12]. Additionally, the association between abnormal BP and adverse outcomes is consistent with findings from other studies [5,13].

Biochemical parameters also play a crucial role in predicting adverse outcomes in preeclampsia. Our study highlighted the significance of abnormal uric acid levels in predicting adverse maternal outcomes, corroborating findings from previous research [9,10]. Furthermore, the association between abnormal fetal Doppler findings and adverse fetal outcomes is consistent with existing evidence [13-15].

Comparing the performance of the fullPIERS model in predicting adverse outcomes with similar studies conducted in India and other countries reveals mixed findings [16-18]. While some studies have reported high sensitivity and specificity of the fullPIERS model in predicting adverse outcomes, others have noted limitations in its predictive accuracy, particularly in certain populations [19].

The study demonstrates the effectiveness of the fullPIERS model in predicting adverse maternal outcomes in preeclampsia, enabling timely and targeted interventions. Implementing this model in clinical practice can improve decision-making, resource allocation, and ultimately, maternal health outcomes, particularly in developing countries where healthcare disparities are prominent.

The study's limitations include a small sample size from a single hospital, limiting generalizability. Potential confounding factors and selection bias due to purposive sampling also affect the validity of the results.

Future research should validate the fullPIERS model in larger, multicenter studies and through longitudinal designs to better understand its long-term impact. Integrating the model with other predictive tools can enhance risk assessment accuracy. Qualitative studies on the model's implementation can provide insights for optimizing its clinical use.

## Conclusions

This study contributes valuable insights into the multifaceted nature of preeclampsia and its associated outcomes. The fullPIERS model is a valuable tool for predicting adverse maternal outcomes in preeclampsia, facilitating timely clinical decision-making. Emphasizing the importance of early identification and management, our study underscores the need for continued research to validate and expand upon our findings. Ultimately, by refining risk assessment strategies and implementing targeted interventions, we can strive toward improving outcomes for mothers and babies affected by preeclampsia.
